# Effectiveness, safety, and practicality of intramuscular progesterone in restoring pregnancy outcomes during frozen embryo transfers

**DOI:** 10.3389/fendo.2025.1592956

**Published:** 2025-05-26

**Authors:** Chien Ngoc Nguyen, Trang Thi Nhu Nguyen, Thanh Thien Le, Phuong Bich Vu, Lan Thi Phuong Le, Nha Ba Pham, Huong Minh To, Xuyen Thi Nguyen, Ly Thi Tam Nguyen, Van Thi Cam Nguyen, Yen Thi Kim Pham, Tran Hue Tran, Quynh Thi Nguyen, Yen Hoang Nguyen, Tho Thi Vu, Thuy Thi Thu Nguyen, Xuan-Hung Nguyen, Robert J. Norman

**Affiliations:** ^1^ Assisted Reproductive Technology Center, Vinmec Healthcare System, Hanoi, Vietnam; ^2^ Vinmec-VinUni Institute of Immunology, College of Health Science, VinUniversity, Hanoi, Vietnam; ^3^ Hi-Tech Center, Vinmec Healthcare System, Hanoi, Vietnam; ^4^ Robinson Research Institute and Adelaide Medical School, University of Adelaide, Adelaide, SA, Australia

**Keywords:** frozen embryo transfer, hormone replacement treatment, luteal phase support, serum progesterone levels, progesterone administration

## Abstract

**Objective:**

To evaluate whether progesterone (P4) supplementation in patients with low serum progesterone levels after normal luteal phase support (LPS) improves pregnancy outcomes in women undergoing frozen embryo transfer (FET).

**Methods:**

A retrospective, single-center cohort study including 1301 vitrified-warmed frozen embryo transfer (FET) cycles conducted from May 2021 to July 2023 at Vinmec Times City International Hospital. A total of 696 FET cycles from individual patients were analyzed for pregnancy outcome. Patients with serum P4 level ≥ 10 ng/ml (Normal P4, n=359) continued to receive standard LPS, while those with P4 levels < 10 ng/ml (Rescue P4, n=337) received 50 mg intramuscular P4 injection daily in addition to the standard LPS for 7–10 days.

**Results:**

Daily intramuscular P4 effectively restored serum P4 levels to that of the Normal P4 group and resulted in pregnancy outcome comparable to that of patients with P4 levels ≥10 ng/ml. Notably, the efficacy of this protocol was consistent even in very low serum P4 concentrations (< 4ng/ml) and was independent of preimplantation genetic testing for aneuploidy (PGT-A).

**Conclusion:**

Intramuscular P4 effectively increased serum P4 levels resulting in pregnancy and neonatal outcomes comparable to those in patients with P4 levels ≥10 ng/ml. This approach provides a safe, effective intervention to improve FET outcomes, supporting the routine inclusion of serum P4 testing and intramuscular P4 rescue in standard FET protocols.

## 1 Introduction

Infertility is recognized by the World Health Organization as a global public health issue (1), with rising incidence particularly in Asia (2, 3). Assisted reproductive technology (ART), notably *in-vitro* fertilization (IVF), has resulted in the birth of over 8 million babies (4). Despite these successes, the live birth rate (LBR) per initiated IVF cycle remains relatively low (5), highlighting the need for more accessible and effective fertility treatments worldwide, especially as the demand for IVF continues to grow.

Frozen embryo transfer (FET) has seen a significant rise over the past decade (6), accounting for 38% of all IVF cycles in 2018 (7). FET classically employs a programmed cycle, which is favored for its ability to achieve pregnancy outcomes comparable to those of natural cycles while also offering the ease of monitoring and more flexible timing of embryo transfer (8, 9). This approach also facilitates elective single-embryo transfer and reduces risks associated with ovarian hyperstimulation syndrome, low birth weight, preterm birth, and perinatal mortality (6, 10, 11).

Hormone replacement therapy (HRT) is integral of FET, simulating endocrine conditions of the menstrual cycle. Exogenous Estrogen is administered from the first or second day of the menstrual cycle for 10–28 days to promote endometrial proliferation, followed by timely administration of exogenous progesterone (P4) (Luteal phase support – LPS). Various routes – oral, vaginal, intramuscular, or subcutaneous injections and various doses of P4 are administered to stimulate the secretory phase (12). Traditionally, intramuscular P4 injections have been used to elevate serum P4 levels and have been shown to improve pregnancy outcomes (13). However, vaginal P4 is increasingly favored due to its ability to bypass the liver’s first-pass metabolism, reducing the risk of thromboembolic complications and achieving higher tissue levels (14, 15). Studies have shown that vaginal P4 achieves similar pregnancy outcomes to intramuscular P4 (16–18), making it a popular choice due to convenience (13).

Until recently, standard doses of P4 administration were the standard care for the luteal phase; however, the individualization of LPS in FET cycles has become the center of attention over the last few years (19). In HRT-FET protocols, suboptimal pregnancy outcomes persist in some programmed FET cycles, often linked to low serum P4 levels (20–22). Low P4 levels on the day of FET are consistently linked to poorer pregnancy outcomes, suggesting that adequate P4 levels are essential for successful implantation and pregnancy maintenance (14, 22, 23). Labarta et al. reported that patients with serum P4 levels below 8.8 ng/ml had significantly lower LBR (35.5% vs 52.0%) (22). Similarly, Cédrin-Durnerin et al. found that patients with P4 levels below 10 ng/ml had a marked reduction in LBR (17% vs 31%) (14), while Gaggiotti-Marre et al. showed that P4 levels below 10.64 ng/ml were associated with lower pregnancy rates (47.5% vs 62.3%) (20). Overall, the literature suggests that serum P4 levels between 8.75 ng/ml to 10.6 ng/ml represent a critical threshold, below which pregnancy outcomes are reduced (14, 21, 22, 24–27). While variability across populations and clinical protocols makes it challenging to define a precise P4 cutoff, 10 ng/ml is widely accepted as a threshold for P4 rescue, as it reflects the levels typically produced by the corpus luteum during a natural cycle (24, 28).

Various strategies have been explored to address low P4 levels. Cédrin-Durnerin and co-workers and others have found that doubling the vaginal P4 dosage on the day of FET did not improve reproductive outcomes, likely due to the rate-limited absorption by the vaginal epithelium (14, 24, 29). This has led to investigations of alternative routes such as intramuscular or subcutaneous P4 injections (19, 30, 31). Studies by Gao et al. and Ozcan et al. demonstrated that P4 injections in patients with P4 levels below 10 ng/ml on the day of FET effectively normalize pregnancy outcomes (30, 32). However, the optimal route, dosage, and frequency of P4 administration remain subjects of ongoing debate, particularly in Asian populations where data are limited.

To bridge this gap, we compared pregnancy outcomes of women in Vietnam undergoing FET with low luteal P4 levels (<10 ng/ml) who received daily 50 mg intramuscular P4, with those in women with normal luteal P4 levels (>10 ng/ml). This study aims to provide critical insights into effective fertility treatments for this high-need population.

## 2 Materials and methods

### 2.1 Study design and patient characteristics

This retrospective study included 1301 vitrified-warmed single FET cycles using standard HRT conducted from May 2021 to July 2023 at the ART Center, Vinmec Times City International Hospital. Patients aged 18–45 years who underwent frozen blastocyst transfers were included. Exclusion criteria were cycles involving oocyte/embryo donation, natural or mild endometrium preparation cycles, multiple embryo transfers, cleavage stage embryo transfers, thin endometrium (<7 mm), or missing serum hormone data on the day of FET. Additionally, cycles employing preimplantation genetic testing for aneuploidy (PGTA) included only euploid embryos, excluding those with aneuploidy or mosaicism. The primary outcome was live birth, defined as the delivery of a baby with signs of life beyond 22 weeks gestation. Secondary outcomes included clinical pregnancy (presence of a gestational sac), ongoing pregnancy (>12 weeks gestation), gestational age at birth, birth weight, and pregnancy loss (the spontaneous demise of a pregnancy, either through miscarriage or stillbirth (33). Sample size calculated to detect non-inferiority in ongoing pregnancy rates between the P4 Rescue and Normal P4 groups, following established guidelines (34). Based on historical data from our center, the baseline ongoing pregnancy rate for FET was estimated at 50%. Using a non-inferiority margin of 10%, with 80% power and a one-sided alpha of 0.05, a minimum sample size of 310 patients per group was required. The study protocol was approved by Vinmec-VinUni Ethical Review Board (96/2024/CN/HDDD VMEC), and informed consent was waived due to the retrospective nature of the study.

### 2.2 IVF process, blastocyst grading, vitrification, and thawing

The IVF procedure was performed according to standard routine protocol. In short, ovarian stimulation was performed using a gonadotropin-releasing hormone agonist/antagonist suppression. Embryos were cultured to the blastocyst stage on Day 5–6 after intracytoplasmic sperm injection. Blastocyst morphology was graded using a modified version of the Gardner and Schoolcraft scoring system (35), based on expansion, inner cell mass and trophectoderm grades. The overall blastocyst morphology was classified as excellent (AA/AB), average (BA/BB), poor (CA/AC/BC/CB/CC), and very poor (all other classifications). Blastocysts categorized as excellent, average, and poor were vitrified and thawed following the manufacturer protocols (Cryotec, Japan) that were previously described by Kuwayama et al (36).

### 2.3 Endometrial preparation and luteal phase support

**Figure 1:**
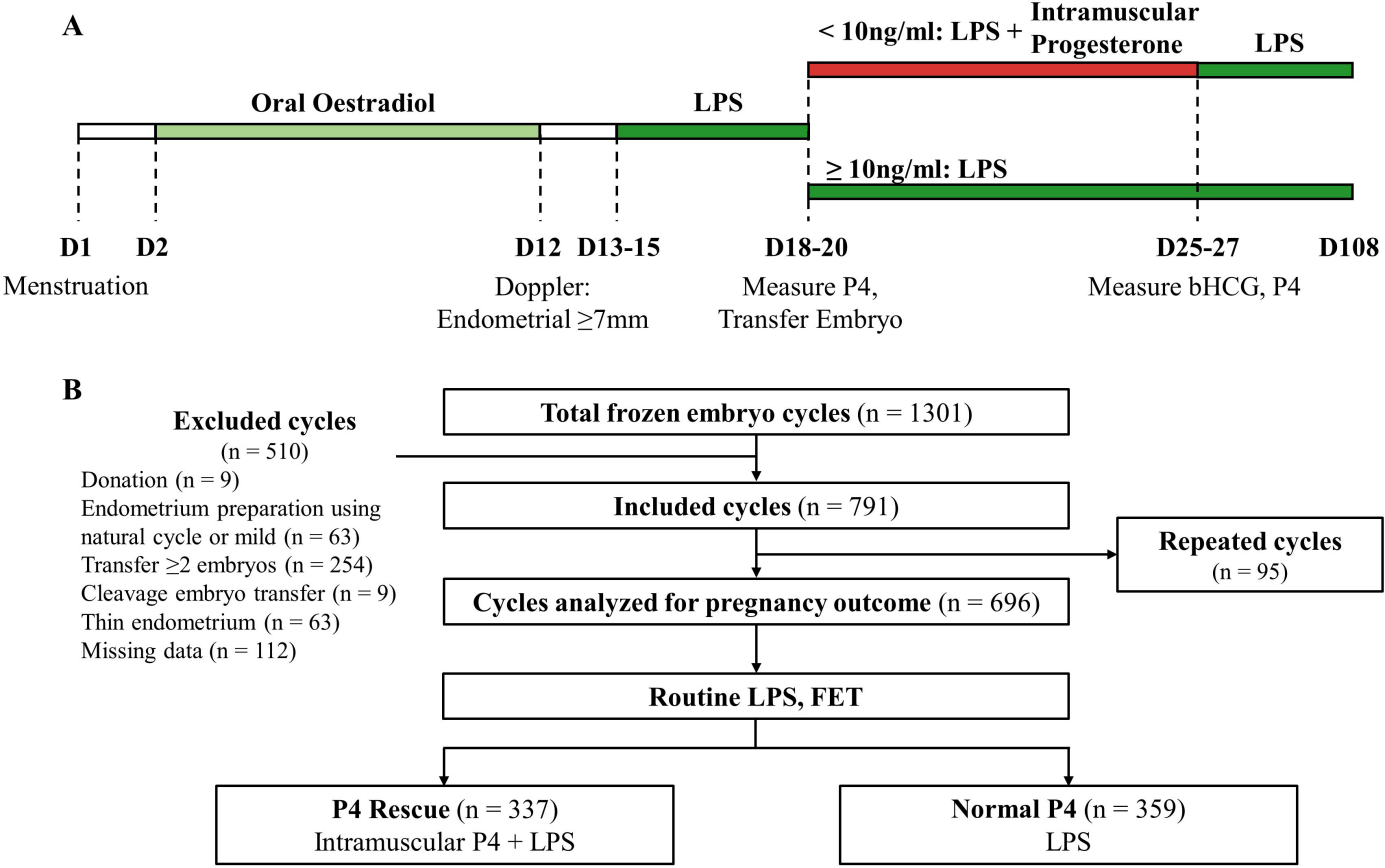
Overall schematics for the study. **(A)** Timeline of LPS, serum P4 measurement and intramuscular P4 rescue. **(B)** Flowchart summarizing the screening and enrollment of this study.

Endometrium was prepared using a standard HRT protocol (**Figure 1A**). Patients received 6–8 mg of oral oestradiol valerate (Valiera, Abbott, Singapore) daily from Day 2 to 12. Doppler ultrasound was performed to assess endometrial thickness and morphology (37). Luteal phase support (LPS) was administered with 400mg vaginal micronized P4 (Utrogestan, Besins Manufacturing Belgium) and 10 mg oral dydrogesterone (Duphaston, Abbott Biological B.V.) twice daily from Day 13-15. Embryo transfer was scheduled 5–6 days after LPS initiation, which was maintained until week 12 of pregnancy.

### 2.4 P4 measurement and intramuscular rescue

Serum P4 measurements were standardized to occur 12 hours after the last dose of vaginal P4. Patients were instructed to take their final vaginal P4 pill at 8:00 PM the evening prior to embryo transfer. Blood samples were then collected at 8:00 AM the following morning, on the day of embryo transfer, to assess serum P4 levels. Patients were classified into two groups: those with P4 levels ≥10 ng/ml continued LPS as previously described (Normal P4 group), while those with P4 levels <10 ng/ml received an additional 50 mg intramuscular P4 (Progesterone Injection BP 25 mg, Rotexmedica GmbH, Germany) daily, alongside standard LPS (P4 Rescue group). Intramuscular supplementation was planned for 10 days, after which both β-hCG and serum P4 levels were reassessed. However, due to patient availability and clinic scheduling, some individuals received treatment and follow-up measurements earlier, with a minimum treatment duration of 7 days. In such cases, if serum P4 remained below 10 ng/mL, daily intramuscular supplementation was continued until completion of the 10-day course.

### 2.5 Statistical analysis

Statistical analysis was conducted using SPSS version 27 (SPSS Inc., IL, USA) and GraphPad Prism version 9 (GraphPad Software, CA, USA). The chi-square test was utilized to compare frequency outcomes between groups, with a two-tailed asymptotic p-value < 0.05 considered statistically significant. Differences were presented as odds ratio with 95% confidence intervals (CI). A t-test was employed to compare means between groups, while a paired t-test was applied to assess differences in progesterone levels of the same patients across multiple FET cycles. A two-tailed p-value < 0.05 was deemed statistically significant. Pearson correlation was used to assess relationships between factors influencing pregnancy outcomes. The Kolmogorov-Smirnov test was employed to analyze distribution differences, considering p-values < 0.05 as statistically significant.

## 3 Results

1301 FET cycles were screened between May 2021 and July 2023. After excluding cycles involving donated embryos, natural cycles, multiple embryo transfers, thin endometrium, and cases with missing data, 791 cycles were included in the study. Subsequently, an additional 95 cycles from patients undergoing transfers with more than one embryo at our facility were excluded from pregnancy outcome analysis to ensure data integrity, resulting in a final dataset comprising 696 cycles representing one patient each (**Figure 1B**). All patients received standard LPS before FET. 359 patients had normal serum P4 level (≥ 10 ng/ml) and continued to receive standard LPS. 337 patients had low P4 level (< 10 ng/ml) and received intramuscular P4 injection in addition to the standard LPS. No patients in the study reported experiencing any adverse side effects from the intramuscular P4 injections.

Between the two cohorts, a small but statistically significant difference was observed in BMI (mean difference = 0.5, p = 0.014), and as expected, a significant difference was noted in serum P4 levels on the day of embryo transfer (mean difference = 10.4 ng/ml, p < 0.001) (**Table 1**). No other differences that could potentially affect FET outcomes were detected, including age, fertility types, endometrial thickness, blastocyst quality, and PGTA usage.

**Table 1:** Baseline patient characteristics of the P4 rescue group versus the normal P4 group.

Characteristic	P4 rescue (n=337)	Normal P4 (n=359)	P value
Female age (years)	32.7 ± 4.3	32.9 ± 4.4	ns
BMI	21.7 ± 2.6	21.2 ± 2.6	0.013
Duration of infertility (years)	3.2 ± 2.5	3.4 ± 2.6	ns
No of IVF attempts (times)	1.5 ± 0.9	1.6 ± 1.0	ns
Type of infertility			ns
Primary	91 (27.0%)	110 (30.6%)	
Secondary	246 (73.0%)	249 (69.4%)	
Indication of IVF			ns
Tubal	61 (18.1%)	57 (15.9%)	
Male	45 (13.4%)	50 (13.9%)	
Unexplained	51 (15.1%)	53 (14.8%)	
Ovulatory	53 (15.7%)	52 (14.5%)	
Mixed	17 (5.0%)	24 (6.7%)	
Others (diminished ovarian reserve, advanced maternal age, history of miscarriage, etc)	110 (32.6%)	123 (34.3%)	
Serum progesterone concentration on embryo transfer day (ng/ml)	6.9 ± 2.1	17.3 ± 10.1	< 0.001
Endometrial thickness (mm)	8.9 ± 1.3	8.9 ± 1.4	ns
Blastocyst quality			ns
Excellent	225 (66.8%)	249 (69.4%)	
Average	65 (19.3%)	75 (20.9%)	
Poor	47 (13.9%)	35 (9.7%)	
PGT-A			0.06
Yes	177 (52.5%)	163 (45.4%)	
No	160 (47.5%)	196 (54.6%)	

Values are expressed as mean ± SD for continuous variables and n (%) for categorical variables.

Binary logistic regression analysis with LBR as the dependent variable identified increased endometrial thickness (OR 1.19, 95% CI 1.06-1.34), higher blastocyst quality (OR 1.82, 95% CI 1.43-2.30), and PGTA usage (OR 2.18, 95% CI 1.56-3.06) as associated with higher odds of live birth (**Supplementary Table 1**). Importantly, due to the administration of intramuscular P4 rescue, serum P4 level was not identified as a predictor of LBR in this analysis, consistent with previous findings (25–27).

Pearson’s correlation analysis revealed no significant correlation between serum P4 levels and age, BMI, infertility duration, type, indication and number of IVF attempts (**Supplementary Figure 1A**). More importantly, serum P4 levels on the day of FET were not correlated with key independent predictors of LBR, including endometrial thickness, blastocyst quality, and PGTA usage. These findings indicate that P4 levels at the time of embryo transfer are independent of other known factors that influence the FET success. Therefore, measuring P4 levels should be considered essential in FET protocols, regardless of the presence or absence of other factors affecting FET outcomes.

**Figure 2:**
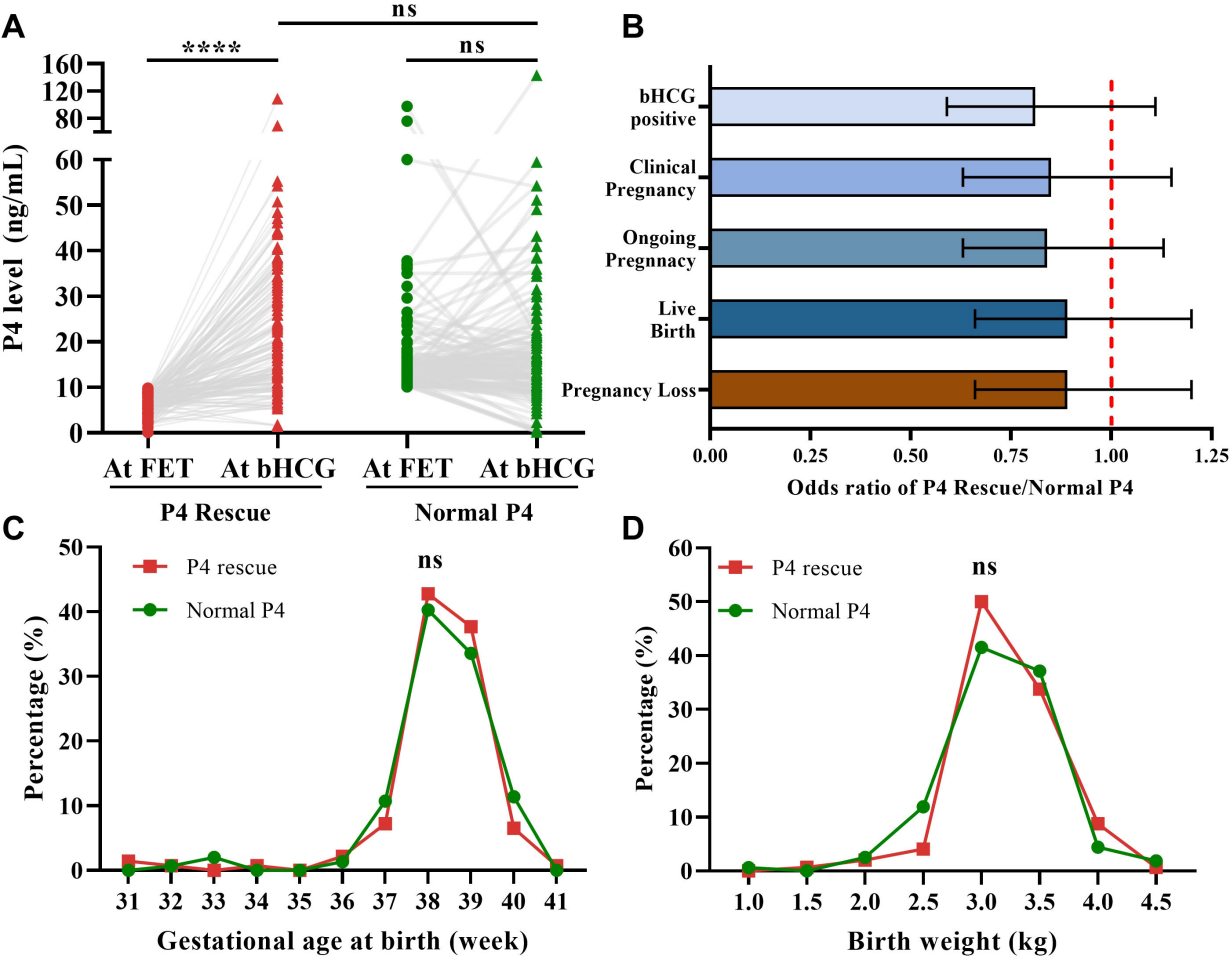
Intramuscular P4 effectively rescues P4 levels and pregnancy outcomes. **(A)** Intramuscular P4 rescue for 7–10 days increased the serum P4 level to normative value (P4 Rescue: n=151, Normal P4: n=122). **(B)** Odds ratio of pregnancy outcomes showed that patients with P4 + Rescue is non-inferior to patients with Normal P4. Error bars denoting 95% CI, (n=696). **(C)** Distribution of delivery term across P4 Rescue and Normal P4 group. **(D)** Distribution of birth weight across P4 Rescue and Normal P4 group, (n=148). ns: p>0.05, ****p <0.001.

Daily intramuscular P4 injection for the P4 Rescue group significantly increased serum P4 levels after 10 days (**Figure 2A**). These elevated P4 levels were comparable to those in the Normal P4 group, suggesting that intramuscular P4 rescue effectively restores normative P4 levels in FET cycles. The primary and secondary outcomes in the P4 Rescue group were comparable to those in the Normal P4 group (**Supplementary Table 2** and **Figure 2B**). The odds ratio for LBR in the P4 rescue group compared to the normal P4 group was 0.89, with a 95% CI of 0.66-1.20. Similarly, the odds ratios for β-hCG positivity, clinical pregnancy, ongoing pregnancy, and pregnancy loss rates were 0.81 (95% CI 0.59-1.11), 0.85 (95% CI 0.63-1.15), 0.84 (95% CI 0.63-1.13), and 0.89 (95% CI 0.66-1.20), respectively. Additionally, in FET cycles that resulted in live births, gestational age and birth weight were similar between the P4 Rescue and Normal P4 groups, both in mean values and distribution (**Figures 2C, D**). These findings indicate that intramuscular P4 rescue not only restores serum P4 levels but also aligns pregnancy outcomes, including live birth rate, gestational age, and birth weight, to those of patients with normal P4 levels.

**Figure 3:**
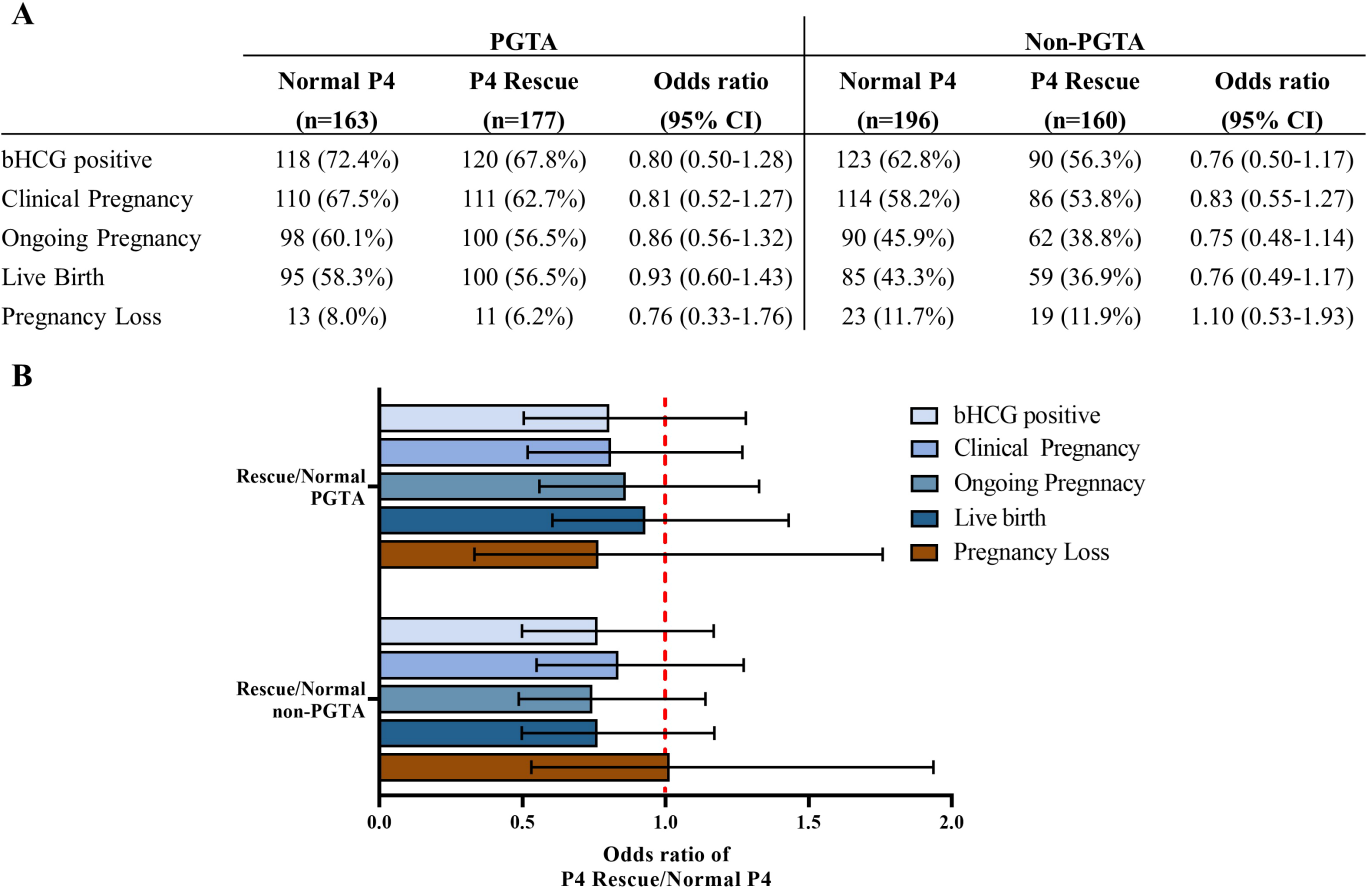
Intramuscular P4 rescue normalizes pregnancy outcomes regardless of PGTA usage. **(A)** Pregnancy and birth outcomes of the P4 Rescue versus the normal P4 group, regarding PGTA usage. **(B)** Odds ratio of pregnancy outcomes showed that patients with P4 Rescue is non-inferior to patients with Normal P4 regarding PGTA usage. Error bars denoting 95% CI.

The rate of PGT-A usage was higher in the P4 Rescue group compared to the Normal P4 group, though this difference was not statistically significant (**Table 1**). Since PGT-A significantly improves pregnancy outcomes and was identified as an independent predictor of LBR in our data (**Supplementary Table 1**), we conducted a subgroup analysis based on PGT-A status (**Figure 3A**). No significant differences in LBR were found between the P4 Rescue and Normal P4 groups, whether PGTA was used or not, with odds ratios of 0.8 (95% CI 0.5-1.28) and 0.7 (95% CI 0.5-1.28), respectively. Similarly, no significant differences in β-hCG positivity, clinical pregnancy, ongoing pregnancy, or pregnancy loss were observed between the groups, regardless of PGT-A usage (**Figure 2B**). These findings suggest that intramuscular P4 rescue normalizes pregnancy outcomes regardless of PGT-A status.

Previous studies have shown a concentration-dependent relationship between serum P4 levels and pregnancy outcomes (20). For example, P4 levels below 5 ng/ml have been reported to result in poor LBR (7.55%) even with rescue and are deemed unsalvageable with P4 rescue (31). To explore this further, we performed a subgroup analysis with serum P4 stratified into 2 ng/ml intervals. Aside from a lower LBR in FET cycles with P4 levels between 18–20 ng/ml compared to 16–18 ng/ml, no significant differences in LBR were observed across other subgroups (**Supplementary Figure 2**). These results indicate that our intramuscular P4 rescue protocol effectively restores pregnancy outcomes across a range of P4 levels below 10 ng/ml.

In summary, our data show that serum P4 is an independent factor influencing FET outcomes. The intramuscular P4 rescue protocol successfully raises P4 levels to the normal range and normalizes pregnancy outcomes, including live birth rates, gestational age, and birth weight. These effects persist even when analyzed in subgroups based on PGTA usage and different serum P4 levels.

## 4 Discussion

In this retrospective, single-center cohort study, we analyzed data from 1301 single FET cycles to assess the efficacy of a daily 50 mg intramuscular P4 injection for patients with serum P4 levels below 10 ng/ml after LPS on the day of embryo transfer. Our findings indicate that intramuscular P4 effectively restored serum P4 levels to normative values and yielded pregnancy outcomes (live birth) and neonatal outcomes (gestational age at birth and birth weight) comparable to those of patients with P4 levels ≥ 10 ng/ml.

Our study has several notable strengths. First, the cohort provides sufficient statistical power to detect significant differences in pregnancy outcomes. The dropout rate was minimal, and P4 supplementation was consistently administered by healthcare providers, ensuring protocol adherence. Secondly, bias was minimized as serum P4 levels were independent of clinical factors and common cofounders such as repeated FET cycles from the same patients and PGTA usage. Our subgroup analysis suggested that the protocol remains effective even for patients with very low serum P4 levels (P4 <4ng/ml), reducing the need for cycle cancellation. However, the sample size for this subgroup was small (n=38); thus, additional studies are required to confirm these preliminary findings. The accessibility and familiarity of intramuscular P4 among healthcare providers further support its practical application in routine clinical practice.

However, several limitations still remain. The retrospective design precluded the inclusion of a control group of patients with low P4 levels who did not receive P4 rescue. Given the well-documented association between low serum P4 and reduced pregnancy chances, ethical considerations prevented us from withholding supplementation from patients with low serum P4, leading to reliance on the commonly accepted cutoff of 10 ng/ml (25, 26). Indirect comparisons from external studies demonstrate significantly lower pregnancy rate in untreated patients with low P4 levels, reporting reduction of 24 - 46% (14, 22, 23). Although national data remain limited and FET protocols vary between facilities, two Vietnamese studies have also reported pregnancy rate reductions of approximately 7–27% in patients with serum P4 below thresholds of 10.9 ng/mL and 9.2 ng/mL, respectively (38). Furthermore, while the optimal cutoff for this population is unknown, subgroup analysis shows that pregnancy outcomes were consistent across all P4 concentrations, suggesting no at-risk groups were missed. Another limitation is the use of oral dydrogesterone (Duphaston), which is not fully detectable by serum P4 assays. However, oral dydrogesterone was only used as an adjunct to vaginal P4 in our protocol, thus its impact on overall P4 activity should be limited. Lastly, while intramuscular P4 is less convenient, the rescue protocol lasted only 7–10 days, with home healthcare options available, contributing to the low dropout rate.

### 4.1 Serum P4 at FET day and pregnancy outcomes

In our study, 48.4% of patients had serum P4 levels below the 10 ng/ml threshold on the day of FET, a higher prevalence higher than the 25% to 37% reported in other Caucasian cohorts (14, 22, 26, 31), but comparable to the 50.5% seen in a Chinese cohort (25). Additionally, 9.1% of our patients had very low serum P4 levels (<5 ng/ml), which has been linked to significantly lower pregnancy outcomes by Volovsky et al. (31). This high prevalence of low P4 levels may suggest racial and geographical differences in P4 absorption or metabolism. Although factors like age, weight, and infertility history have been suggested to influence P4 levels (39), our analysis showed no significant association between serum P4 levels and these clinical variables, including BMI, endometrial thickness, and even consecutive FET cycles in the same patients. These findings highlight the need for further investigation into population-specific and protocol-specific factors that affect P4 absorption and bioavailability. The independence of serum P4 to other clinical factors underscores the critical role in pregnancy outcomes, reinforcing the importance of routine P4 assessment during FET.

### 4.2 LPS P4 rescue protocol

Given the importance of serum P4 levels in FET success, significant efforts have been made to enhance LPS by supplementing patients with P4 before or during embryo implantation. However, due to variability across populations and ART centers, identifying a universally optimal protocol remains challenging. Researchers have explored various approaches to P4 rescue, with both subcutaneous and intramuscular routes showing effective outcomes. For example, Alvarez et al. demonstrated that administering 25 mg of subcutaneous P4 daily to patients with low P4 levels before FET achieved pregnancy outcomes comparable to controls (24). Similarly, Yarali et al. and Ozcan et al. reported that daily subcutaneous 25 mg P4 effectively normalized P4 levels and supported successful pregnancies (26, 27). Further, the different administration routes may cause P4 to reach the endometrium with different efficacy and therefore cause a variable exposure.

Despite the efficacy of subcutaneous P4, intramuscular P4 is sometimes preferred due to its robust track record in clinical practice. Studies, such as those by Gao et al., showed that 40 mg of intramuscular P4 daily significantly improved outcomes for patients with low serum P4 levels compared to standard LPS (25). Devine et al. expanded this approach by administering intramuscular P4 to all patients, regardless of serum P4 levels, and found that adding intramuscular P4 to LPS significantly enhanced pregnancy outcomes (40).

The timing of P4 measurement is another crucial factor. While Alvarez et al. suggested measuring P4 the day before blastocyst transfer (24), recent studies advocate for assessing serum P4 on the day of embryo transfer, as it more accurately reflects the endometrium’s receptivity (25, 26). Additionally, the timing of rescue P4 treatment is vital. Delcour et al. found no improvement in outcomes when intramuscular P4 was administered after the β-hCG test, stressing the importance of early intervention (41).

Concerns about elevated progesterone levels negatively impacting pregnancy outcomes have been recently addressed. Cozzolino et al. in a large retrospective analysis of 7,546 FET cycles, demonstrated no difference in ongoing pregnancy rates, live birth rates, or miscarriage rates across groups with serum progesterone concentrations of >40 ng/ml, 20–40 ng/ml, and <20 ng/ml (42). Similarly, Firat Tulek et al. found that progesterone levels exceeding 60 ng/ml did not adversely influence pregnancy outcomes (43). These findings align closely with our results, where patients with serum P4 >20 ng/ml showed comparable LBR, suggesting that P4 supplementation leading to elevated serum concentrations after embryo transfer can be considered safe and effective. Nevertheless, optimizing progesterone dosage and duration remains crucial for patient convenience and cost-effectiveness.

Our clinical protocol involves measuring serum progesterone on the day of FET and administering 50 mg intramuscular progesterone daily, alongside standard LPS, to patients with P4 <10 ng/ml. This approach, requiring minimal testing, results in LBR and neonatal outcomes comparable to those of patients with normal P4 levels. The widespread availability of intramuscular P4, ease of administration by healthcare providers, and its proven safety profile, with no significant side effects, make it a preferable option for improving clinical outcomes in FET protocols.

## 5 Conclusion

Our retrospective study demonstrates that intramuscular progesterone effectively restores serum P4 levels and ensures pregnancy and neonatal outcomes comparable to those observed in patients with adequate P4 levels on the day of frozen embryo transfer. Despite limitations inherent in the retrospective design, including the absence of an untreated low-progesterone control group, our findings align with existing literature, underscoring the safety and efficacy of intramuscular P4 injection. Given the high prevalence of low serum P4 levels in our cohort, possibly reflecting population-specific pharmacokinetics and genetics, routine P4 measurement and intramuscular P4 rescue should be considered essential components of clinical practice. Further prospective studies, particularly focused on patients with extremely low progesterone levels, are warranted to refine protocols and optimize clinical outcomes.

## Data Availability

The original contributions presented in the study are included in the article/**Supplementary Material**. Further inquiries can be directed to the corresponding author/s.

## References

[1] Sexual and Reproductive Health and Research (SRH). Infertility prevalence estimates 1990–2021. Geneva, Switzerland: WHO (2023). Available online at: https://www.who.int/publications/i/item/978920068315.

[2] Borumandnia N Alavi Majd H Khadembashi N Alaii H. Worldwide trend analysis of primary and secondary infertility rates over past decades: A cross-sectional study. Int J Reprod BioMed. (2022) 20:37–46. doi: 10.18502/ijrm.v20i1.10407 35308328 PMC8902794

[3] Borumandnia N Majd HA Khadembashi N Alaii H. Assessing the trend of infertility rate in 198 countries and territories in last decades. Iran J Public Health. (2021) 50:1735–7. doi: 10.18502/ijph.v50i8.6840 PMC8643527 34917549

[4] Kamath MS Mascarenhas M Franik S Liu E Sunkara SK. Clinical adjuncts in *in vitro* fertilization: a growing list. Fertil Steril. (2019) 112:978–86. doi: 10.1016/j.fertnstert.2019.09.019 31703943

[5] Saket Z Källén K Lundin K Magnusson Å Bergh C. Cumulative live birth rate after IVF: trend over time and the impact of blastocyst culture and vitrification. Hum Reprod Open. (2021) 2021:hoab021. doi: 10.1093/hropen/hoab021 34195386 PMC8240131

[6] Singh B Reschke L Segars J Baker VL. Frozen-thawed embryo transfer: the potential importance of the corpus luteum in preventing obstetrical complications. Fertil Steril. (2020) 113:252–7. doi: 10.1016/j.fertnstert.2019.12.007 PMC7380557 32106972

[7] Human Fertilisation & Embryology Authority. Fertility treatment 2018: trends and figures (2018). Available online at: https://www.hfea.gov.uk/about-us/publications/research-and-data/fertility-treatment-2018-trends-and-figures/ (Accessed June 5, 2024).

[8] Agha-Hosseini M Hashemi L Aleyasin A Ghasemi M Sarvi F Shabani Nashtaei M. Natural cycle versus artificial cycle in frozen-thawed embryo transfer: A randomized prospective trial. Turk J Obstet Gynecol. (2018) 15:12–7. doi: 10.4274/tjod.47855 PMC5894530 29662710

[9] Pelinck MJ Hoek A Simons AHM Heineman MJ. Efficacy of natural cycle IVF: a review of the literature. Hum Reprod Update. (2002) 8:129–39. doi: 10.1093/humupd/8.2.129 12099628

[10] Ginström Ernstad E Wennerholm U-B Khatibi A Petzold M Bergh C. Neonatal and maternal outcome after frozen embryo transfer: Increased risks in programmed cycles. Am J Obstetrics Gynecology. (2019) 221:126.e1–126.e18. doi: 10.1016/j.ajog.2019.03.010 30910545

[11] Sha T Yin X Cheng W Massey IY. Pregnancy-related complications and perinatal outcomes resulting from transfer of cryopreserved versus fresh embryos *in vitro* fertilization: a meta-analysis. Fertil Steril. (2018) 109:330–342.e9. doi: 10.1016/j.fertnstert.2017.10.019 29331236

[12] Groenewoud ER Cohlen BJ Macklon NS. Programming the endometrium for deferred transfer of cryopreserved embryos: hormone replacement versus modified natural cycles. Fertility Sterility. (2018) 109:768–74. doi: 10.1016/j.fertnstert.2018.02.135 29778369

[13] Yanushpolsky EH. Luteal phase support in *in vitro* fertilization. Semin Reprod Med. (2015) 33:118–27. doi: 10.1055/s-0035-1545363 25734349

[14] Cédrin-Durnerin I Isnard T Mahdjoub S Sonigo C Seroka A Comtet M. Serum progesterone concentration and live birth rate in frozen-thawed embryo transfers with hormonally prepared endometrium. Reprod BioMed Online. (2019) 38:472–80. doi: 10.1016/j.rbmo.2018.11.026 30642638

[15] Miles RA Paulson RJ Lobo RA Press MF Dahmoush L Sauer MV. Pharmacokinetics and endometrial tissue levels of progesterone after administration by intramuscular and vaginal routes: a comparative study. Fertil Steril. (1994) 62:485–90. doi: 10.1016/s0015-0282(16)56935-0 8062942

[16] Kaser DJ Ginsburg ES Missmer SA Correia KF Racowsky C. Intramuscular progesterone versus 8% Crinone vaginal gel for luteal phase support for day 3 cryopreserved embryo transfer. Fertil Steril. (2012) 98:1464–9. doi: 10.1016/j.fertnstert.2012.08.007 22959457

[17] Shapiro DB Pappadakis JA Ellsworth NM Hait HI Nagy ZP. Progesterone replacement with vaginal gel versus i.m. injection: cycle and pregnancy outcomes in IVF patients receiving vitrified blastocysts. Hum Reprod. (2014) 29:1706–11. doi: 10.1093/humrep/deu121 PMC4093993 24847018

[18] Tournaye H Sukhikh GT Kahler E Griesinger G. A Phase III randomized controlled trial comparing the efficacy, safety and tolerability of oral dydrogesterone versus micronized vaginal progesterone for luteal support in *in vitro* fertilization. Hum Reprod. (2017) 32:1019–27. doi: 10.1093/humrep/dex023 PMC5400051 28333318

[19] Mumusoglu S Polat M Ozbek IY Bozdag G Papanikolaou EG Esteves SC. Preparation of the endometrium for frozen embryo transfer: A systematic review. Front Endocrinol. (2021) 12:688237. doi: 10.3389/fendo.2021.688237 PMC8299049 34305815

[20] Gaggiotti-Marre S Martinez F Coll L Garcia S Álvarez M Parriego M. Low serum progesterone the day prior to frozen embryo transfer of euploid embryos is associated with significant reduction in live birth rates. Gynecol Endocrinol. (2019) 35:439–42. doi: 10.1080/09513590.2018.1534952 30585507

[21] Labarta E Mariani G Paolelli S Rodriguez-Varela C Vidal C Giles J. Impact of low serum progesterone levels on the day of embryo transfer on pregnancy outcome: a prospective cohort study in artificial cycles with vaginal progesterone. Hum Reprod. (2021) 36:683–92. doi: 10.1093/humrep/deaa322 33340402

[22] Labarta E Mariani G Holtmann N Celada P Remohí J Bosch E. Low serum progesterone on the day of embryo transfer is associated with a diminished ongoing pregnancy rate in oocyte donation cycles after artificial endometrial preparation: a prospective study. Hum Reprod. (2017) 32:2437–42. doi: 10.1093/humrep/dex316 29040638

[23] Shekhar B Mittal S Majumdar G Tiwari N Majumdar A. Low serum progesterone on day of transfer adversely impacts ongoing pregnancy rates in hormonally prepared single blastocyst frozen embryo transfer cycles. Eur J Obstet Gynecol Reprod Biol. (2023) 289:55–9. doi: 10.1016/j.ejogrb.2023.08.016 37639815

[24] Álvarez M Gaggiotti-Marre S Martínez F Coll L García S González-Foruria I. Individualised luteal phase support in artificially prepared frozen embryo transfer cycles based on serum progesterone levels: a prospective cohort study. Hum Reprod. (2021) 36:1552–60. doi: 10.1093/humrep/deab031 33686413

[25] Gao H Ye J Ye H Hong Q Sun L Chen Q. Strengthened luteal phase support for patients with low serum progesterone on the day of frozen embryo transfer in artificial endometrial preparation cycles: a large-sample retrospective trial. Reprod Biol Endocrinol. (2021) 19:60. doi: 10.1186/s12958-021-00747-8 33892741 PMC8063468

[26] Ozcan P Cetin C Okten B Tanoglu FB Taha HS Pasin O. The importance of serum progesterone concentration at embryo transfer day and effect of rescue additional progesterone during programmed artificial frozen embryo transfer cycles. Reprod BioMed Online. (2022) 45:785–92. doi: 10.1016/j.rbmo.2022.05.023 35810079

[27] Yarali H Polat M Mumusoglu S Ozbek IY Erden M Bozdag G. Subcutaneous luteal phase progesterone rescue rectifies ongoing pregnancy rates in hormone replacement therapy vitrified-warmed blastocyst transfer cycles. Reprod BioMed Online. (2021) 43:45–51. doi: 10.1016/j.rbmo.2021.04.011 34016521

[28] Hull MG Savage PE Bromham DR Ismail AA Morris AF. The value of a single serum progesterone measurement in the midluteal phase as a criterion of a potentially fertile cycle (“ovulation”) derived form treated and untreated conception cycles. Fertil Steril. (1982) 37:355–60. doi: 10.1016/s0015-0282(16)46095-4 7060786

[29] Paulson RJ Collins MG Yankov VI. Progesterone pharmacokinetics and pharmacodynamics with 3 dosages and 2 regimens of an effervescent micronized progesterone vaginal insert. J Clin Endocrinol Metab. (2014) 99:4241–9. doi: 10.1210/jc.2013-3937 24606090

[30] Stavridis K Kastora SL Triantafyllidou O Mavrelos D Vlahos N. Effectiveness of progesterone rescue in women presenting low circulating progesterone levels around the day of embryo transfer: a systematic review and meta-analysis. Fertility Sterility. (2023) 119:954–63. doi: 10.1016/j.fertnstert.2023.02.007 36781098

[31] Volovsky M Pakes C Rozen G Polyakov A. Do serum progesterone levels on day of embryo transfer influence pregnancy outcomes in artificial frozen-thaw cycles? J Assist Reprod Genet. (2020) 37:1129–35. doi: 10.1007/s10815-020-01713-w PMC7244647 32043182

[32] Melo P Chung Y Pickering O Price MJ Fishel S Khairy M. Serum luteal phase progesterone in women undergoing frozen embryo transfer in assisted conception: a systematic review and meta-analysis. Fertility Sterility. (2021) 116:1534–56. doi: 10.1016/j.fertnstert.2021.07.002 34384594

[33] Kolte AM Bernardi LA Christiansen OB Quenby S Farquharson RG Goddijn M. Terminology for pregnancy loss prior to viability: a consensus statement from the ESHRE early pregnancy special interest group. Hum Reprod. (2015) 30:495–8. doi: 10.1093/humrep/deu299 25376455

[34] Piaggio G Elbourne DR Altman DG Pocock SJ Evans SJW CONSORT Group. Reporting of Noninferiority and Equivalence Randomized TrialsAn Extension of the CONSORT Statement. . JAMA. (2006) 295:11521160. doi: 10.1001/jama.295.10.1152 16522836

[35] Gardner DK Schoolcraft WB. Culture and transfer of human blastocysts. Curr Opin Obstet Gynecol. (1999) 11:307–11. doi: 10.1097/00001703-199906000-00013 10369209

[36] Kuwayama M Vajta G Kato O Leibo SP. Highly efficient vitrification method for cryopreservation of human oocytes. Reprod BioMed Online. (2005) 11:300–8. doi: 10.1016/s1472-6483(10)60837-1 16176668

[37] Wang L Qiao J Li R Zhen X Liu Z. Role of endometrial blood flow assessment with color Doppler energy in predicting pregnancy outcome of IVF-ET cycles. Reprod Biol Endocrinol. (2010) 8:122. doi: 10.1186/1477-7827-8-122 20955593 PMC2974732

[38] Vuong LN Pham TD Le KTQ Ly TT Le HL Nguyen DTN. Micronized progesterone plus dydrogesterone versus micronized progesterone alone for luteal phase support in frozen-thawed cycles (MIDRONE): a prospective cohort study. Human Reproduction . (2021) 36:18211831. doi: 10.1093/humrep/deab093 33930124

[39] Maignien C Bourdon M Marcellin L Guibourdenche J Chargui A Patrat C. Clinical factors associated with low serum progesterone levels on the day of frozen blastocyst transfer in hormonal replacement therapy cycles. Hum Reprod. (2022) 37:2570–7. doi: 10.1093/humrep/deac199 36125015

[40] Devine K Richter KS Jahandideh S Widra EA McKeeby JL. Intramuscular progesterone optimizes live birth from programmed frozen embryo transfer: a randomized clinical trial. Fertil Steril. (2021) 116:633–43. doi: 10.1016/j.fertnstert.2021.04.013 33992421

[41] Delcour C Robin G Delesalle A-S Drumez E Plouvier P Dewailly D. Weekly intramuscular progesterone for luteal phase support in women receiving oocyte donation is associated with a decreased miscarriage rate. Reprod BioMedicine Online. (2019) 39:446–51. doi: 10.1016/j.rbmo.2019.05.001 31311693

[42] Cozzolino M Hervás I Ergun Y Massaro MG Pellicer N de Angelis F. Higher serum progesterone level has no negative impact on live birth rate in frozen embryo transfer. Eur J Obstetrics Gynecology Reprod Biol. (2024) 303:15–21. doi: 10.1016/j.ejogrb.2024.10.011 39395245

[43] Dr, Firat T Pabuccu E Pabuccu R Demirel C. VERY HIGH SERUM PROGESTERONE LEVELS ON THE DAY OF EMBRYO TRANSFER DO NOT HAVE AN IMPACT ON OUTCOMES IN ARTIFICIALLY PREPARED FROZEN EMBRYO TRANSFER CYCLES. Fertility Sterility. (2022) 118:e162. doi: 10.1016/j.fertnstert.2022.08.468

