# Supplementary material for: Effectiveness, safety, and practicality of intramuscular progesterone in restoring pregnancy outcomes during frozen embryo transfers
Source: Front Endocrinol (Lausanne). 2025 May 26;16:1592956. doi: 10.3389/fendo.2025.1592956 (PMC12146201; doi:10.3389/fendo.2025.1592956)
Supplement: Supplementary file 1 [file DataSheet1.docx]

**Optimizing Frozen Embryo Transfer: Intramuscular progesterone Rescue for Low Serum Progesterone Levels**

**Supplementary Information**


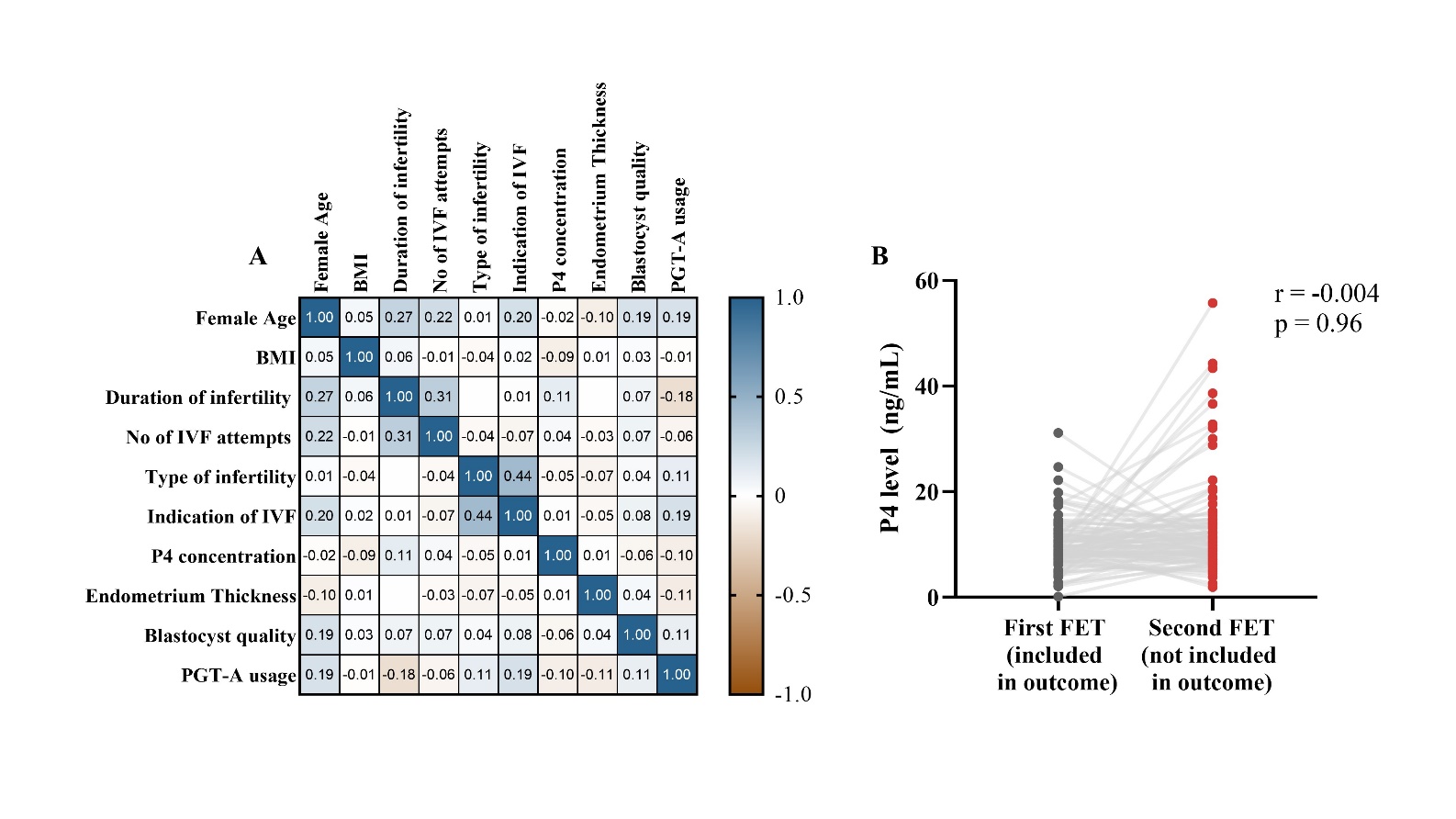


**Supplementary Figure 1. Serum P4 level at the day of FET is an independent factor affecting pregnancy outcomes. A)** P4 level is independent of factors such as age, BMI, infertility year, IVF attempts, endometrium thickness, blastocyst quality and PGTA usage. **B)** Serum P4 concentration of different FET cycles from the same patients are independent.

**Supplementary Table 1: Logistic regression to identify independent predictors of ongoing pregnancy in P4 Rescue versus Normal P4**

| **Characteristic** | **OR (95%CI)** | **P value** |
| --- | --- | --- |
| Female age | 0.99 (0.95 - 1.03) | 0.585 |
| BMI | 1.018 (0.96 - 1.08) | 0.569 |
| Duration of infertility | 0.97 (0.90 - 1.04) | 0.333 |
| No of IVF attempts | 0.91 (0.76 - 1.08) | 0.293 |
| Type of infertility | 1.06 (0.70 - 1.60) | 0.783 |
| Indication of IVF | 0.96 (0.89 - 1.05) | 0.432 |
| Serum progesterone level | 1.01 (0.99 - 1.03) | 0.343 |
| Endometrium thickness | 1.18 (1.05 - 1.33) | **0.005** |
| Blastocyst quality* | 1.84 (1.44 - 2.35) | **<0.001** |
| PGT-A** | 2.34 (1.61 - 3.42) | **<0.001** |

*Odd ratio of excellent blastocyst quality/ poor blastocyst quality

** Odd ratio of FET cycles with PGT-A/ cycles without

**Supplementary Table 2. Pregnancy and birth outcomes of the P4 Rescue versus the normal P4 group**

|  | **P4 Rescue** | **Normal P4** | **Odds Ratio** |
| --- | --- | --- | --- |
|  | **(n=337)** | **(n=359)** | **(95% CI)** |
| **bHCG positive** | 210 (62.3%) | 241 (67.1%) | 0.81 (0.59-1.11) |
| **Clinical Pregnancy** | 197 (58.5%) | 224 (62.4%) | 0.85 (0.63-1.15) |
| **Ongoing Pregnancy** | 162 (48.1%) | 188 (52.4%) | 0.84 (0.63-1.13) |
| **Live Birth** | 159 (47.2%) | 180 (50.1%) | 0.89 (0.66-1.20) |
| **Pregnancy Loss** | 30 (8.9%) | 36 (10.0%) | 0.89 (0.66-1.20) |


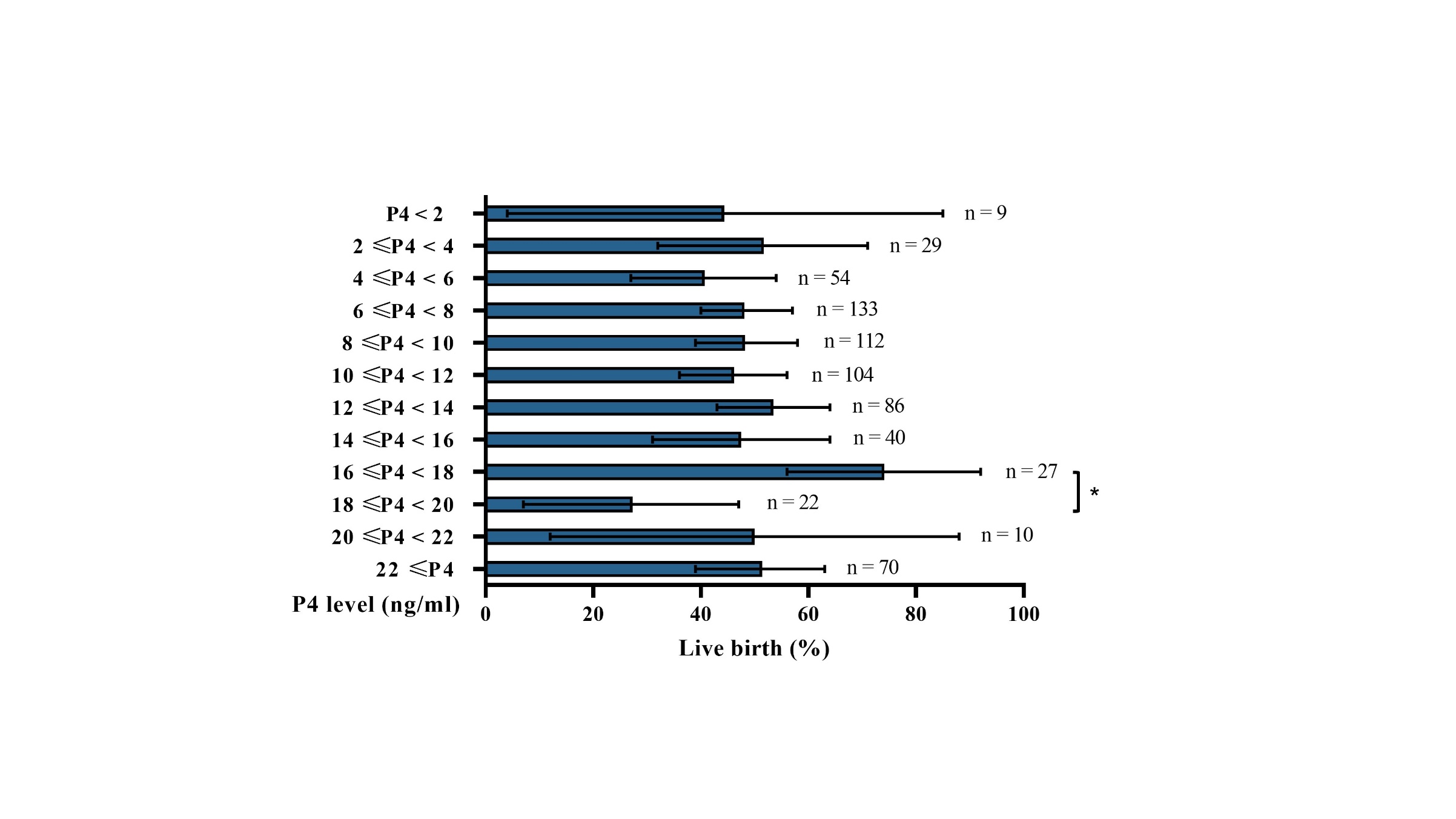


**Supplementary Figure 2: Pregnancy outcomes are not significantly different between patient subgroups based on serum P4 level.** Error bars denoting 95% CI. *: p<0.05
